# Acute Performance and Velocity-Based Fatigue Responses to Alternated and Grouped Exercise Orders in Full-Body Circuit Resistance Training

**DOI:** 10.3390/sports14040141

**Published:** 2026-04-03

**Authors:** Francisco Hermosilla-Perona, Adrián Martín-Castellanos, Marcos R. Pereira-Monteiro, Javier Iglesias García, Manuel Barba-Ruíz, Juan R. Heredia-Elvar

**Affiliations:** 1 Facultad de Ciencias Biomédicas y de la Salud, Universidad Alfonso X El Sabio, Villanueva de la Cañada, 28691 Madrid, Spain; 2 Facultad de Ciencias de la Vida y la Naturaleza, Universidad Nebrija, 28015 Madrid, Spain; 3 Facultad de Ciencias de la Actividad Física y del Deporte, Sports Department, Universidad Politécnica de Madrid, 28040 Madrid, Spain; 4 Graduate Program in Physiological Sciences, Federal University of Sergipe, São Cristóvão 49100-000, SE, Brazil

**Keywords:** strength, exercise, force, squat, bench press

## Abstract

Introduction: Circuit resistance training is widely used to enhance physical performance. However, the acute-performance- and fatigue-related effects of exercise order and volume in circuit training, particularly between upper and lower limbs, remain unclear. Objectives: This study examined acute velocity-based responses to different exercise orders and volumes during full-body circuit resistance training. Methods: Thirty resistance-trained adults completed four circuit protocols: alternating exercises with maximal repetitions per exercise (A1), grouped exercises with maximal repetitions per exercise (G1), alternating exercises with 50% of maximal repetitions in the first round (A2), and grouped exercises with 50% of maximal repetitions in the first round (G2). Mean propulsive velocity (MPV) in the bench press and squat at 60% 1RM was assessed before and after each circuit. Results: A significant main effect of Time was observed for both bench press and squat MPV (*p* < 0.001), with no Intervention × Time interactions. Alternating configurations showed larger effect sizes, indicating greater velocity loss. Under equal volume, upper limbs exhibited greater performance decline than lower limbs. Conclusions: Although exercise order did not result in statistically significant differences, alternating configurations induced a greater magnitude of fatigue-related performance decline than grouped configurations, particularly in upper-body exercises.

## 1. Introduction

In resistance training, controlling the variables of the exercise dose is essential to understand the effects of the training. Among the ways of verifying the effects of the dose is the acute performance response to resistance exercise, which informs about the body’s immediate reaction to stimulus and can be modulated by variables such as the number of repetitions and the interval between sets [1]. One of the variables that can be manipulated to modify the training dose is the methodological organization of exercises, which can be structured using horizontal or vertical training models. In horizontal models, all sets of a given exercise are completed before moving on to the next exercise, thereby grouping sets with the same movement pattern [2]. In contrast, vertical models organize exercises within the same set, such that different exercises are performed sequentially with short rest intervals, as commonly occurs in circuit training [3].

Comparisons between these different models for ordering circuit training have been highlighted in the literature [2,4]. Some differences in chronic adaptations have been found: models that did exercises sequentially and with the same motor pattern had better adaptations in isometric strength and girdle stability compared to exercises that were sequential and had an alternating motor pattern [5]. In addition to the different chronic effects, exercises performed in different configurations also seem to have different acute responses. When grouping exercises with the same muscle group, training can lead to larger muscle damage compared to alternating the muscle groups targeted in the exercises [6]. When organizing exercises in a circuit model, it is common practice to divide exercises into upper- and lower-limb categories. In grouped models, exercises for upper and lower limbs are performed sequentially (e.g., upper, upper, lower, lower). On the other hand, in the alternating model, exercises are performed such that they alternate between upper and lower limbs (e.g., upper, lower, upper, lower) [7].

Although some studies have compared dose components in different circuit-training models, it should be noted that the comparisons performed involve endurance training models [4,8], specific populations [9,10] or environmental conditions [11]. As far as we know, fewer studies have specifically addressed the acute responses associated with exercise order in resistance-based circuit training. Regarding this, Corrêa Neto, do Nascimento Silva, Palma, de Oliveira, Vingren, Marchetti, da Silva Novaes and Monteiro [7] researched the effects of grouped and alternating circuit models of resistance training with a focus on the number of repetitions and volume load. However, the variable used to determine the intensity of the protocol exercises was based on maximum repetition (RM). This is a limited variable for reliably expressing the intensity—velocity of movement is a more reliable variable for assessing it—and thus the changes in the force production in strength exercises [12,13]. In this sense, lower velocities indicates a closer proximity to maximal repetition (RM) capacity. Consequently, larger reductions in mean propulsive velocity (MPV) during a set reflect greater fatigue accumulation and a higher level of fatigue [14].

Furthermore, recent evidence suggests that minor adjustments in training variables, including exercise order, may significantly alter acute performance and fatigue-related effects [7]. Consequently, systematically examining exercise order within full-body circuit resistance training is necessary to better understand how the methodological organization of exercises influences training dose and the magnitude of fatigue induced by different circuit configurations. In this context, it is also important to compare fatigue-related performance responses between upper- and lower-limb exercises, as these segments may respond differently to the same training volume during circuit-based training. Identifying such differences is relevant for ensuring that comparable training stimuli are applied to both segments. From a practical perspective, this knowledge can help optimize exercise sequencing, training density, and effort regulation within a single training session. In this regard, reductions in MPV can be used as an indicator of fatigue accumulation during a session, with greater decreases reflecting higher levels of fatigue. As an indicator, MPV may help professionals involved in resistance training prescription (e.g., strength and conditioning coaches and exercise professionals) adjust exercise order or volume to better manage fatigue during circuit-based resistance training. Moreover, understanding these acute responses is important because the stimuli generated during individual training sessions ultimately contribute to the chronic adaptations produced by resistance training programs.

This study aimed to understand how different exercise orders and volumes in full-body circuit resistance training affect acute performance and velocity-based responses. It was hypothesized that grouped exercise order would induce greater acute declines in performance, reflected by larger reductions in MPV, compared with alternated exercise order.

## 2. Materials and Methods

### 2.1. Design

We conducted an experimental crossover study to examine the acute responses to different training configurations (alternated and grouped) and different predefined volume conditions. We used the bench press and squat with 60% of one-repetition maximum (1RM) and continued until participants reached muscular failure or submaximal performance according to MPV. Bench press and squat exercises were selected as representative upper- and lower-body multi-joint movements due to their widespread use in resistance training practice and their suitability for standardized and reliable MPV assessment [12]. MPV was used as an indirect, performance-based marker of fatigue-related changes in force production [14].

All participants visited the laboratory four times to complete the experimental sessions. A familiarization session was conducted before the experimental tests.

### 2.2. Participants

Thirty young adults participated in the study (weight: 67.93 ± 14.14 kg; height: 1.60 ± 0.32 m; age: 22.48 ± 4.58 years). Participants comprised 19 men and 11 women (18–30 years) with a minimum of one year of experience in resistance training, categorized as recreationally trained by Rhea [15]. Participants were excluded if they reported any injury during the study period or within the previous six months.

Participants were fully briefed on the experimental procedures and potential risks before participation, and written informed consent was obtained from all participants. The study protocol received approval from the local ethics committee (approval number: 2024_12/312).

The recruitment period for the study was from January 2025 to March 2025.

### 2.3. Procedures

In the study design, five sessions were conducted, each separated by 48–72 h. Each session involved specific exercises and a specific workload organization. In all sessions, the exercises performed were three upper-limb exercises (bench press (BP), shoulder press (SP), and bilateral rowing (BR)) and three lower-limb exercises ((leg press (LP), leg curl (LC), and leg extension (LE)). The selection of exercises was influenced by equipment availability and logistical constraints within the laboratory setting. Nevertheless, exercise choice was guided by the premise of including representative push and pull movements for both the upper and lower limbs in order to reflect a typical full-body circuit resistance training structure. These exercises were executed using guided machines to ensure a correct technical execution and load adaptation.

During each session, subjects completed two rounds of the circuit involving these six exercises. The load was determined in the first session (familiarization), in which subjects completed the entire circuit and subjectively established a load that allowed them to perform 10–12 repetitions until failure.

The following sessions were organized into alternating and grouped exercise arrangements. The alternating circuit (A1) mixed upper- and lower-limb exercises, while the grouped circuit (G1) involved performing the three upper-limb exercises first, followed by the three lower limb exercises. In A1 and G1, participants performed the maximum number of repetitions in both rounds for all exercises. To account for the different pre-established volume conditions, in the third and fourth sessions (A2 and G2), the order remained the same as in A1 and G1, respectively, but participants performed half the number of repetitions in the first round as they did in A1 and G1 and were required to perform as many repetitions as possible in the second round. Rest intervals between exercises and between rounds were standardized: participants rested for one minute between exercises and two minutes between rounds. For each round and exercise, repetitions were calculated. Before performing each exercise, participants were asked to estimate the maximum number of repetitions they believed they could complete with the assigned load.

**Figure 1:**
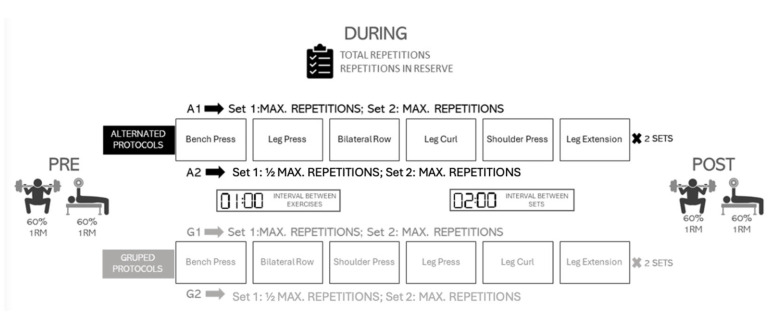
Procedures conducted.

For all participants, MPV during the concentric phase of the bench press and squat was assessed at the beginning (PRE) and at the end (POST) of each circuit to evaluate changes in force production induced by the circuits. During the familiarization session, an incremental loading protocol was performed for the bench press and squat to determine the load corresponding to 60% 1RM. The target load was identified using previously established and validated MPV values associated with ~60% 1RM (≈0.78 m·s^−1^ for the bench press and ≈1.00 m·s^−1^ for the squat) [12]. The data were recorded with a validated linear encoder (Vitruve encoder, Vitruve fit, Madrid, Spain) [16,17]. The distribution of exercises and measurements is explained in Figure 1.

### 2.4. Statistical Analysis

An a priori sample size calculation was conducted using G*Power software (version 3.1.9.2, Kiel, Germany). The calculation was based on a repeated-measures ANOVA design with within-subject factors, assuming a medium effect size (f = 0.25), an alpha level of 0.05, and a statistical power of 0.80. This analysis indicated that a minimum sample size of 27 participants was required. Assumptions of normality were assessed using the Shapiro–Wilk test, sphericity was evaluated using Mauchly’s test, and homoscedasticity was checked through residual analysis. For repetition-related variables, model selection was performed using the Akaike Information Criterion (AIC), in accordance with previous recommendations for longitudinal and repetition-based performance data [18]. All variables included in the analyses were normally distributed (*p* > 0.05); therefore, parametric statistical procedures were applied without the need for data transformation or nonparametric alternatives.

A two-way repeated-measures ANOVA (4 × 2) was conducted to examine the effects of circuit condition (A1, G1, A2, or G2) and time (PRE vs. POST) on MPV in the bench press and squat exercises. When significant main effects or interactions were detected, Bonferroni-adjusted post hoc comparisons were applied. For analyses involving the number of repetitions and estimation error, exercise (BP, BR, SP, LP, LC, and LE) was included as an additional within-subject factor. In these cases, the statistical model consisted of a repeated-measures ANOVA with six exercise levels and four circuit conditions (6 × 4 design).

We adopted a significance level of 0.05 for all the analyses. Effect sizes were calculated using Cohen’s d (d) and classified as small (d = 0.20–0.49), medium (d = 0.50–0.79), or large (d ≥ 0.80). Effect sizes for repeated-measures ANOVA were reported separately as partial eta squared (ηp^2^) and categorized as being above the 0.01, 0.06, or 0.15 thresholds for small, medium, and large effect sizes, respectively [19].

Estimation error was calculated for descriptive purposes only, to provide an indication of the accuracy of participants’ perceived maximum repetition capacity. Participants’ estimated number of repetitions was compared with the actual maximum number of repetitions performed, and estimation error was expressed as a percentage based on the difference between estimated and performed repetitions relative to the performed repetitions.

All the data were analyzed using the statistical software Jamovi (The Jamovi Project, Jamovi, Version 2.4.11), and graphs were built using the GraphPad Prism (version 10.0.0 for Windows, GraphPad Software, Boston, MA, USA, www.graphpad.com).

## 3. Results

**Figure 2:**
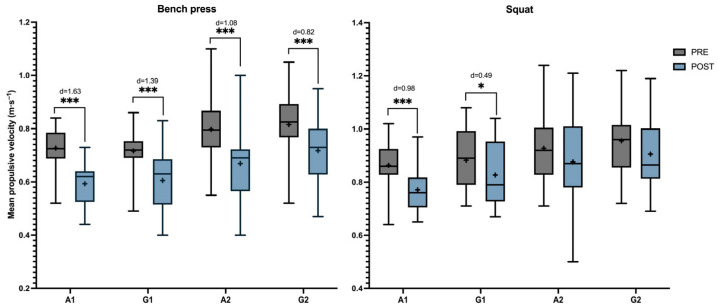
Mean propulsive velocity of different groups across time in bench press and squat. Note: A1—Alternated 1, A2—Alternated 2, G1—Grouped 1, and G2—Grouped 2. Error bars represent minimum to maximum values. Sig codes: * *p* < 0.05, and *** *p* < 0.001. d represents Cohen’s d.

Comparative analyses of before (PRE) and after (POST) session measurements across the different circuit conditions are shown in Figure 2. The ANOVA results for the squat revealed a significant main effect of Time on squat MPV (f (1,29) = 52.74, *p* < 0.001, ηp^2^ = 0.65), indicating a reduction from PRE to POST. No significant main effect of Intervention was observed (f (3,87) = 1.94, *p* = 0.13, ηp^2^ = 0.06), and the Intervention × Time interaction was not significant. Similarly, for the bench press, the ANOVA revealed a significant main effect of Time on MPV (f 1,29) = 238.64, *p* < 0.001, ηp^2^ = 0.89). No significant main effect of Intervention was observed (f (3,87) = 2.31, *p* = 0.082, ηp^2^ = 0.07), and the Intervention × Time interaction was not significant.

Bonferroni post hoc analysis showed that, in the bench press, all circuit conditions presented significant differences between PRE and POST (*p* < 0.001). However, the circuits in which the participants performed the maximal number of repetitions (A1 and G1) presented large and moderate effect sizes (d = 1.63 and d = 1.39 respectively), with greater MPV loss after the protocol than in the circuits with 50% of repetitions performed during the first lap (A2 and G2). In the case of the squat, which showed fewer variations between PRE and POST, exercise did not show the same pattern as the bench press. Only the circuits with the maximal number of repetitions during the two rounds (A1 and G1) showed significant differences (*p* < 0.001 and *p* < 0.05 respectively). A2 and G2 did not report any statistical differences.

**Figure 3:**
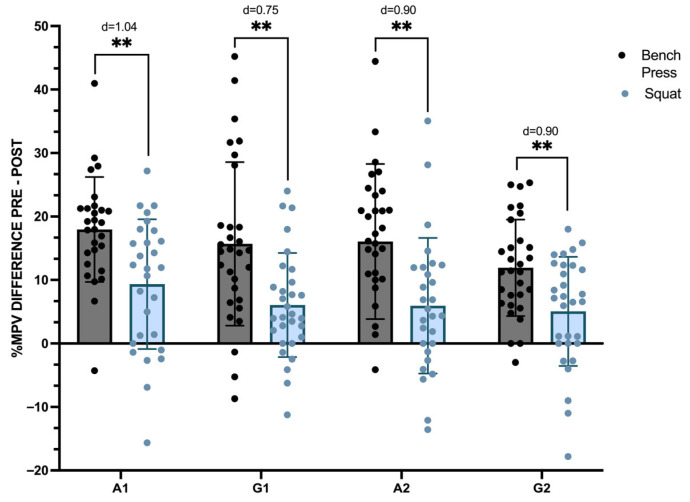
Differences in the groups’ mean propulsive velocity percentages for the bench press and squat. Note: A1—Alternated 1, A2—Alternated 2, G1—Grouped 1, and G2—Grouped 2. Sig codes: ** *p* < 0.01.

Figure 3 presents the percentage difference in MPV from pre-circuit (PRE) to post-circuit (POST) for both the bench press and squat exercises across four circuits (A1, G1, A2, and G2). The ANOVA revealed a significant main effect of Exercise (f (1,29) = 41.86, *p* < 0.001, ηp^2^ = 0.59), indicating greater velocity loss in the bench press compared with the squat. No significant Interaction effect in Exercise × Intervention was found. In the post-hoc analysis of exercises, comparisons revealed that all had a significantly greater percentage loss of MPV for the bench press compared to the squat (*p* < 0.01). Specifically, the descriptive results show that the bench press presents an MPV loss ranging from ~18% to ~12%, contrasting with only a ~10% to 5% loss in the squat exercise. In comparing the circuit protocols, only one comparison displays significant differences. In comparing the bench press between A1 and G2, A1 shows a higher loss of MPV than G2 (*p* < 0.05).

**Figure 4:**
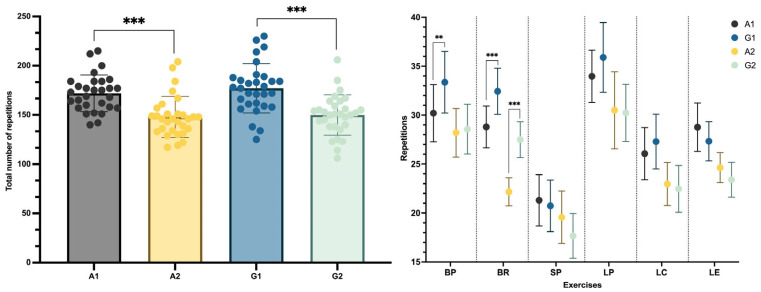
Total number of repetitions overall and by exercise. Note: A1—Alternated 1, A2—Alternated 2, G1—Grouped 1, G2—Grouped 2, BP—bench press, BR—bilateral row, SP—shoulder press, LP—leg press, LC—leg curl, and LE—leg extension Sig codes: ** *p* < 0.01, and *** *p* < 0.001.

Figure 4 provides a descriptive overview of the total number of repetitions performed in each condition (A1, A2, G1, and G2) and across specific exercises, reflecting the predefined volume constraints of each protocol. The two-way repeated-measures ANOVA revealed a significant main effect of Exercise (f (5,145) = 18.74, *p* < 0.001, ηp^2^ = 0.39). No significant main effect of Intervention was observed. However, a significant Exercise × Intervention interaction was found (f (15,435) = 3.41, *p* < 0.001, ηp^2^ = 0.11).

As expected, conditions performed to momentary muscular failure (A1 and G1) showed a significantly higher total number of repetitions compared with conditions in which repetitions were limited during the first round (A2 and G2) (*p* < 0.001). The same pattern was found between G1 and G2 conditions, with higher volumes for G1 (*p* < 0.001). Comparing the exercises showed that condition A1 consistently shows higher total repetitions and repetitions across individual exercises compared to A2, except for the BP. The significant *p*-values (*p* < 0.01) indicate a marked difference in performance volume. In G1 and G2, similar results could be observed, but in this case, the BP also presents differences between the repetitions performed (*p* < 0.001). In comparing A1 and G1, condition G1 generally shows higher repetitions, particularly in exercises like the BP and BR (*p* < 0.01). Finally, comparing A2 and G2 showed that both conditions present a similar amount of repetitions performed, except for the BR, in which G2 presents higher values than A2 (*p* < 0.001).

**Figure 5:**
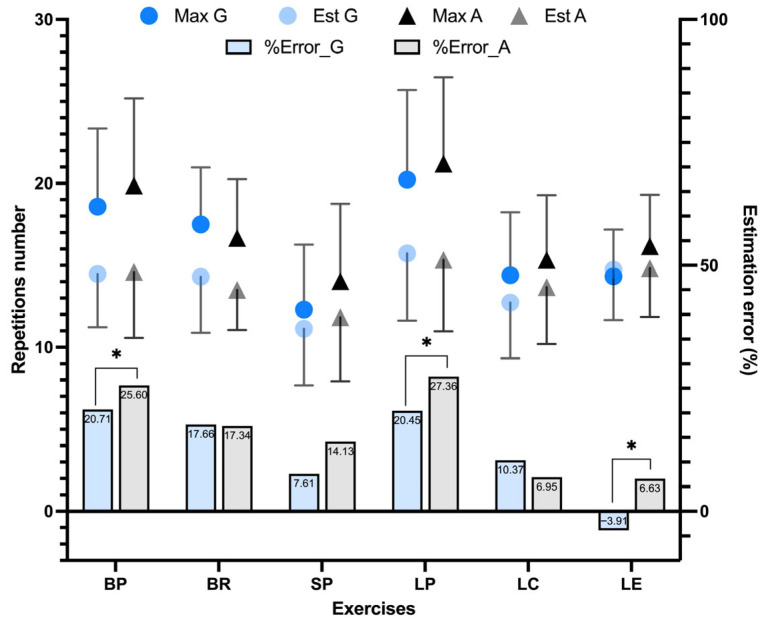
Repetition estimations and performed repetitions during the first and second rounds of the circuits. Note: BP—bench press, BR—bilateral row, SP—shoulder press, LP—leg press, LC—leg curl, and LE—leg extension. Max A—maximal repetitions performed in alternated circuits. Max G—maximal repetitions performed in grouped circuits. Est A—maximal repetitions estimated in alternated circuits. Est G—maximal repetitions estimated in grouped circuits. Sig codes: * *p* < 0.05.

The analysis revealed a significant main effect of Exercise on the repetition estimation error (f (5,145) = 6.82, *p* < 0.001, ηp^2^ = 0.19), indicating differences between exercises. No significant main effect of Intervention was observed, and the Exercise × Intervention interaction was not significant. The post hoc analysis revealed significant differences between the estimation errors for BP, LP, and LE (*p* < 0.05) (Figure 5). These results correspond to conditions A1 and G1, which were used to determine the maximal number of repetitions for each exercise. Grouped circuits generally exhibited lower estimation errors compared to alternated circuits for these exercises. Conversely, for other exercises like BR and LC, alternated circuits demonstrated comparable or slightly lower estimation errors. It could be highlighted that for the multiarticular exercises, like BP or LP, the estimation errors are higher than for the isolated exercises, such as LC, LE and SP.

## 4. Discussion

This study aimed to understand how different exercise orders and volumes in full-body circuit resistance training affect acute performance and velocity-based responses. Specifically, the ordering and distribution of exercises within the circuit were analyzed. Quantifying fatigue-related changes in movement velocity provides insight into how different circuit configurations influence immediate performance response within a session. This information can support decision-making regarding exercise order, volume distribution, and effort regulation, allowing professionals involved in resistance training prescription (e.g., strength and conditioning coaches and exercise professionals) to manage fatigue.

When circuit configurations were compared under equivalent volume conditions (A1 vs. G1), the alternated exercise order (A1) induced a greater decline in acute performance than the grouped configuration (G1), as reflected by larger pre–post reductions in MPV. This greater velocity loss in A1 was evident in both the upper-body (bench press) and lower-body (squat) exercises, indicating a higher fatigue response despite matched training volume. These findings suggest that exercise order may influence the magnitude of fatigue during full-body circuit resistance training. A possible explanation is that alternating exercises targeting different muscle groups may increase the accumulation of systemic fatigue across the circuit due to repeated involvement of large muscle masses and sustained cardiovascular and metabolic demand. This greater systemic stress may negatively affect neuromuscular performance and reduce the capacity to maintain movement velocity in subsequent exercises. Previous research has shown that exercise sequencing and the configuration of resistance training tasks can modulate acute fatigue responses and performance outcomes during multi-exercise sessions [6,7]

Interestingly, when comparing upper- and lower-limb exercises, the magnitude of MPV reduction was consistently greater in the bench press than in the squat, suggesting a more pronounced fatigue response in the upper limbs. This pattern is consistent with previous research reporting greater velocity loss and reductions in force production in upper-body exercises compared with lower-body exercises when performed at comparable relative intensities and effort levels [20,21,22]. Lower limb movements typically involve a larger active muscle mass and greater fatigue resistance, which may attenuate performance decline [20,22]. From a practical perspective, these results suggest that when aiming to produce a comparable training stimulus between upper- and lower-body exercises, the training volume prescribed for lower-limb exercises may need to be reduced, given lower limbs’ greater resistance to fatigue.

When examining the maximum volume achieved across different exercise configurations, significant differences were observed in the actual volume completed, particularly between conditions designed with equivalent prescribed volume (A1 vs. G1). Despite both protocols requiring participants to perform the maximal number of repetitions per exercise, the grouped configuration (G1) resulted in a greater total number of repetitions than the alternated configuration (A1). Notably, this occurred despite the alternated configuration (A1) showing greater reductions in MPV, indicating a greater fatigue and force loss. These results highlight exercise order as a relevant variable for modulating the balance between fatigue and volume in circuit resistance training. However, these results contrast with previous research in which configurations that alternated exercises and modified exercise order reportedly showed no significant differences in total workload or perceived exertion, supporting the notion that exercise sequencing can influence performance outcomes without necessarily altering perceived effort [23].

From an applied perspective, improving knowledge regarding the use of the nature of effort to regulate training load is important for optimizing decision-making in resistance training programs [14,24]. Although the present study was not specifically designed to test the effects of circuit structure on repetition estimation accuracy, the analysis of estimation error (Figure 5) was included as a complementary outcome to support the interpretation of the main results. The present findings indicate that the accuracy with which individuals estimate their maximal repetition capacity was significantly influenced by both exercise type and circuit structure. This is in line with evidence that the maximal number of repetitions achievable at a given relative load varies (REPS-%1RM) substantially across exercises, and that between-individual variability in the REPS-%1RM relationship is considerable [25]. The lower estimation error in grouped configurations may reflect more stable perceptual feedback and fatigue accumulation, enabling a more accurate estimation of proximity to failure. These observations are consistent with previous studies indicating that effort regulation is task-dependent and influenced by exercise complexity and fatigue perception [24], and highlight the need for caution when applying effort-based prescription strategies in circuit-training contexts [26].

These findings provide practical information for exercise professionals designing full-body circuit resistance training sessions. Specifically, the observed differences in fatigue-related performance responses suggest that exercise order can be manipulated to prioritize either fatigue accumulation or repetition volume, depending on the training objective. For instance, alternated configurations may be preferable when a greater acute fatigue stimulus is intended, as they appear to promote a larger accumulation of fatigue within the session. This may be particularly relevant in training contexts where fatigue and metabolic stress are desirable stimuli, such as hypertrophy-oriented resistance training [27,28]. In contrast, grouped configurations may allow a greater repetition volume to be maintained with smaller MPV reductions, which could be advantageous when preserving movement velocity and technical execution throughout the session is a priority, such as in power-oriented resistance training [29,30]. To refine evidence-based circuit-training prescription, future research should further explore how modifying training density or rest interval duration influences these performance-based fatigue responses.

A limitation of the present study is that, due to the guided nature of the circuit-training protocol, load selection and exercise intensity were determined individually and subjectively for each participant. This approach was necessary to preserve the ecological validity of the circuit format and to ensure continuous execution across exercises; however, it limited the possibility of objectively standardizing intensity across all exercises using external metrics such as movement velocity. As a result, inter-individual variability in perceived effort and load selection may have influenced the magnitude of the responses observed. Also, the time between the final exercise performed within the circuit and the POST MPV assessment differed depending on exercise order. In alternated circuits, the elapsed time between the last upper-limb exercise and the bench press MPV test may have been longer than in grouped circuits, potentially allowing partial recovery and influencing the magnitude of velocity loss observed. This factor should be considered when interpreting differences between conditions and warrants further investigation in future studies.

## 5. Conclusions

In summary, circuit structure plays a relevant role in the magnitude of fatigue developed during a session. Specifically, when exercise order was compared under equivalent volume conditions, the alternated configuration produced greater fatigue-related performance decline than the grouped configuration, as reflected by larger reductions in MPV. Notably, the decline in performance was more pronounced in the upper-body exercise (bench press) than in the lower-body exercise (squat), indicating a greater tendency for fatigue-related performance decline in the upper limbs.

From a practical perspective, when exercise order was compared under equivalent volume conditions, the alternated configuration showed greater fatigue-related performance decline than the grouped configuration, as reflected by larger reductions in mean propulsive velocity. These findings highlight the relevance of considering both exercise selection and volume management when designing circuit resistance training programs aimed at controlling fatigue and performance responses.

## Data Availability

The data used in this study were unavailable due to ethical restrictions.

## References

[1] Sánchez-Moreno M., Rodiles-Guerrero L., Rendeiro-Pinho G., Prieto-Veloso A., Pareja-Blanco F. (2023). Acute Mechanical and Metabolic Responses to Different Resistance Training Protocols With Equated Volume Load. Int. J. Sports Physiol. Perform..

[2] Waller M., Miller J., Hannon J. (2011). Resistance circuit training: Its application for the adult population. Strength. Cond. J..

[3] Gettman L.R., Ayres J.J., Pollock M.L., Jackson A. (1978). The effect of circuit weight training on strength, cardiorespiratory function, and body composition of adult men. Med. Sci. Sports.

[4] Nuñez T.P., Amorim F.T., Beltz N.M., Mermier C.M., Moriarty T.A., Nava R.C., VanDusseldorp T.A., Kravitz L. (2020). Metabolic effects of two high-intensity circuit training protocols: Does sequence matter?. J. Exerc. Sci. Fit..

[5] Da Silva-Grigoletto M.E., Neto E.P., Brandão L.H.A., Chaves L.M.D.S., Bezerra de Almeida M. (2020). Efeitos de dois tipos de protocolos de cross training sobre a composição corporal e aptidão física. Rev. Bras. Fisiol. Exerc..

[6] Brentano M.A., Umpierre D., Santos L.P., Lopes A.L., Radaelli R., Pinto R.S., Kruel L.F. (2017). Muscle damage and muscle activity induced by strength training super-sets in physically active men. J. Strength. Cond. Res..

[7] Corrêa Neto V.G., do Nascimento Silva D., Palma A., de Oliveira F., Vingren J.L., Marchetti P.H., da Silva Novaes J., Monteiro E.R. (2024). Comparison Between Traditional and Alternated Resistance Exercises on Blood Pressure, Acute Neuromuscular Responses, and Rating of Perceived Exertion in Recreationally Resistance-Trained Men. J. Strength. Cond. Res..

[8] Contrò V., Bianco A., Cooper J., Sacco A., Macchiarella A., Traina M., Proia P. (2017). Effects of different circuit training protocols on body mass, fat mass and blood parameters in overweight adults. J. Biol. Res.-Boll. Soc. Ital. Biol. Sper..

[9] Freitas T.T., Calleja-González J., Alarcón F., Alcaraz P.E. (2016). Acute effects of two different resistance circuit training protocols on performance and perceived exertion in semiprofessional basketball players. J. Strength. Cond. Res..

[10] Fonseca G.F., Midgley A.W., Billinger S.A., Michalski A.C., Costa V.A., Monteiro W., Farinatti P., Cunha F.A. (2022). Acute effects of mixed circuit training on hemodynamic and cardiac autonomic control in chronic hemiparetic stroke patients: A randomized controlled crossover trial. Front. Physiol..

[11] Ramos-Campo D.J., Rubio-Arias J.Á., Freitas T.T., Camacho A., Jiménez-Diaz J.F., Alcaraz P.E. (2017). Acute physiological and performance responses to high-intensity resistance circuit training in hypoxic and normoxic conditions. J. Strength. Cond. Res..

[12] González-Badillo J.J., Sánchez-Medina L. (2010). Movement velocity as a measure of loading intensity in resistance training. Int. J. Sports Med..

[13] Elvar J.R.H., García-Orea G.P., Muñoz J.L.M.M., Lougedo J.H., De-Oliveira L.A., Da Silva-Grigoletto M.E. (2021). Determinação e controle da intensidade e volume do treinamento de força na pesquisa nas ciências do exercício e sua aplicação. Rev. Bras. Fisiol. Exerc..

[14] Sanchez-Medina L., González-Badillo J.J. (2011). Velocity loss as an indicator of neuromuscular fatigue during resistance training. Med. Sci. Sports Exerc..

[15] Rhea M.R. (2004). Determining the magnitude of treatment effects in strength training research through the use of the effect size. J. Strength. Cond. Res..

[16] González-Galán J., Herrera-Bermudo J.C., González-Badillo J.J., Rodríguez-Rosell D. (2024). Validity and Concordance of a Linear Position Transducer (Vitruve) for Measuring Movement Velocity during Resistance Training. Sensors.

[17] Justo-Álvarez A., García-López J., Sabido R., García-Valverde A. (2025). Validity of a New Portable Sensor to Measure Velocity-Based Resistance Training. Methods Protoc..

[18] Berlanga L., Matos-Duarte M., López-Chicharro J. (2023). Effects of active vs. passive interset rest among physiological and perceptual outcomes in bench press exercise. Sci. Sports.

[19] Cohen J. (1988). Statistical Power Analysis for the Behavioral Sciences.

[20] Rodríguez-Rosell D., Yáñez-García J.M., Sánchez-Medina L., Mora-Custodio R., González-Badillo J.J. (2020). Relationship between velocity loss and repetitions in reserve in the bench press and back squat exercises. J. Strength. Cond. Res..

[21] González-Badillo J.J., Yañez-García J.M., Mora-Custodio R., Rodríguez-Rosell D. (2017). Velocity loss as a variable for monitoring resistance exercise. Int. J. Sports Med..

[22] Izquierdo M., González-Badillo J., Häkkinen K., Ibanez J., Kraemer W., Altadill A., Eslava J., Gorostiaga E.M. (2006). Effect of loading on unintentional lifting velocity declines during single sets of repetitions to failure during upper and lower extremity muscle actions. Int. J. Sports Med..

[23] Pirauá A.L.T., Beltrão N.B., Lima Júnior D.R.A.A.d., Queiroz G.R.d., Souza J.G.d., Melo B.M., Araújo R.C.d. (2014). Efeito da ordem dos exercícios sobre o desempenho durante uma sessão de treinamento resistido no método circuito. Rev. Bras. Cineantropometria Desempenho Hum..

[24] Hernández-Belmonte A., Courel-Ibáñez J., Conesa-Ros E., Martínez-Cava A., Pallarés J.G. (2022). Level of effort: A reliable and practical alternative to the velocity-based approach for monitoring resistance training. J. Strength. Cond. Res..

[25] Nuzzo J.L., Pinto M.D., Nosaka K., Steele J. (2024). Maximal Number of Repetitions at Percentages of the One Repetition Maximum: A Meta-Regression and Moderator Analysis of Sex, Age, Training Status, and Exercise. Sports Med..

[26] Ramos-Campo D.J., Andreu Caravaca L., Martínez-Rodríguez A., Rubio-Arias J. (2021). Effects of Resistance Circuit-Based Training on Body Composition, Strength and Cardiorespiratory Fitness: A Systematic Review and Meta-Analysis. Biology.

[27] Pareja-Blanco F., Rodríguez-Rosell D., Sánchez-Medina L., Sanchis-Moysi J., Dorado C., Mora-Custodio R., Yáñez-García J.M., Morales-Alamo D., Pérez-Suárez I., Calbet J.A.L. (2017). Effects of velocity loss during resistance training on athletic performance, strength gains and muscle adaptations. Scand. J. Med. Sci. Sports.

[28] Krzysztofik M., Wilk M., Wojdała G., Gołaś A. (2019). Maximizing Muscle Hypertrophy: A Systematic Review of Advanced Resistance Training Techniques and Methods. Int. J. Environ. Res. Public Health.

[29] Marshall J., Bishop C., Turner A., Haff G.G. (2021). Optimal Training Sequences to Develop Lower Body Force, Velocity, Power, and Jump Height: A Systematic Review with Meta-Analysis. Sports Med..

[30] Riscart-López J., Sánchez-Valdepeñas J., Mora-Vela R., Caro-Ávalos J., Sánchez-González L., Sánchez-Moreno M., León-Prados J.A., Pareja-Blanco F. (2024). Effects of 4 Different Velocity-Based Resistance-Training Programming Models on Physical Performance. Int. J. Sports Physiol. Perform..

